# Supplementary figures and images for: Genome-Wide Identification and Analysis of Fruit Expression Patterns of the TCP Gene Family in Three Genera of Juglandaceae
Source: Biology (Basel). 2025 Oct 30;14(11):1529. doi: 10.3390/biology14111529 (PMC12649894; doi:10.3390/biology14111529)

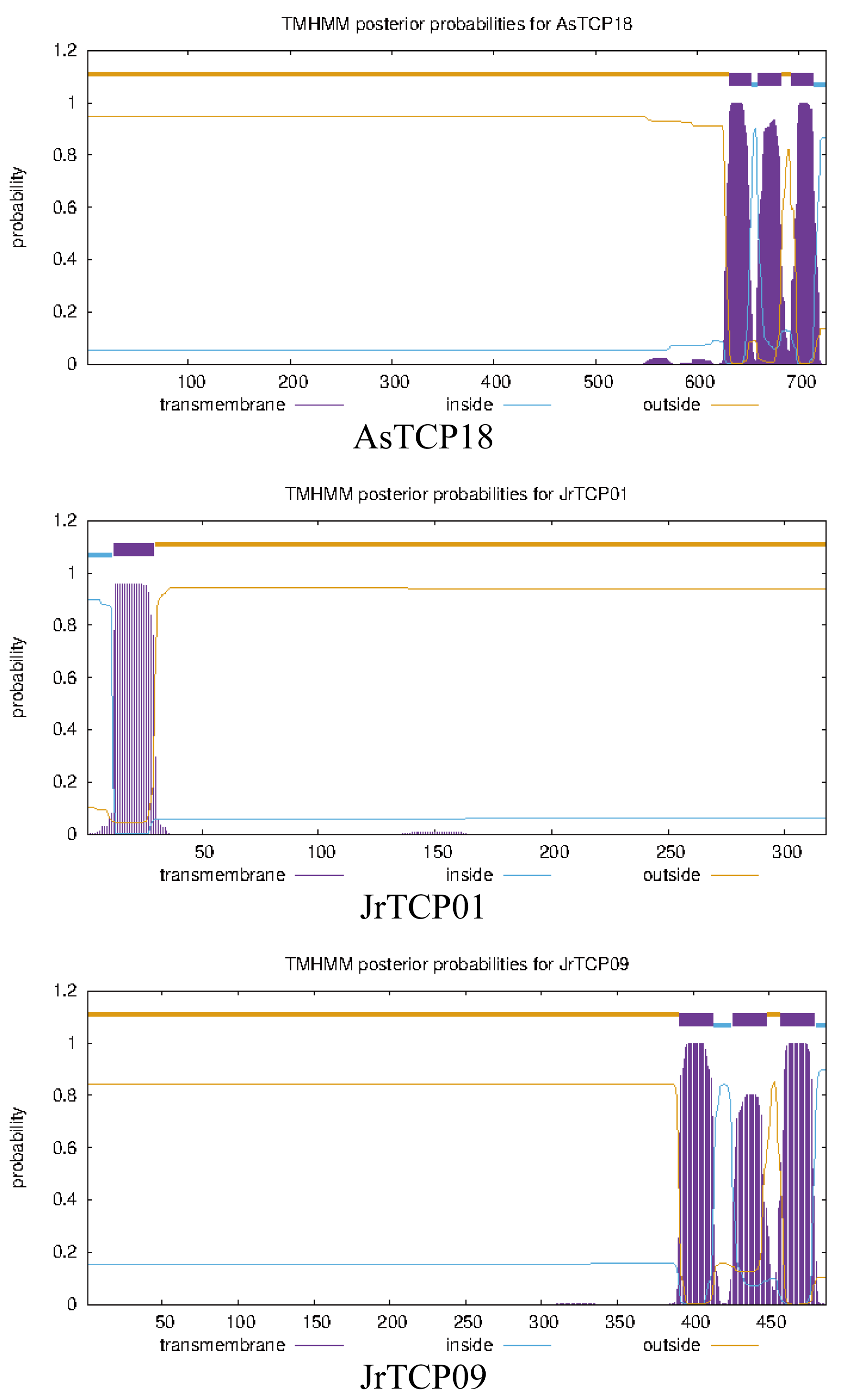

Supplement: Supplementary file 1 [file biology-14-01529-s001.zip › Figure S1.Transmembrane Distribution of AsTCP18, JrTCP01, and JrTCP09 Proteins.tiff]

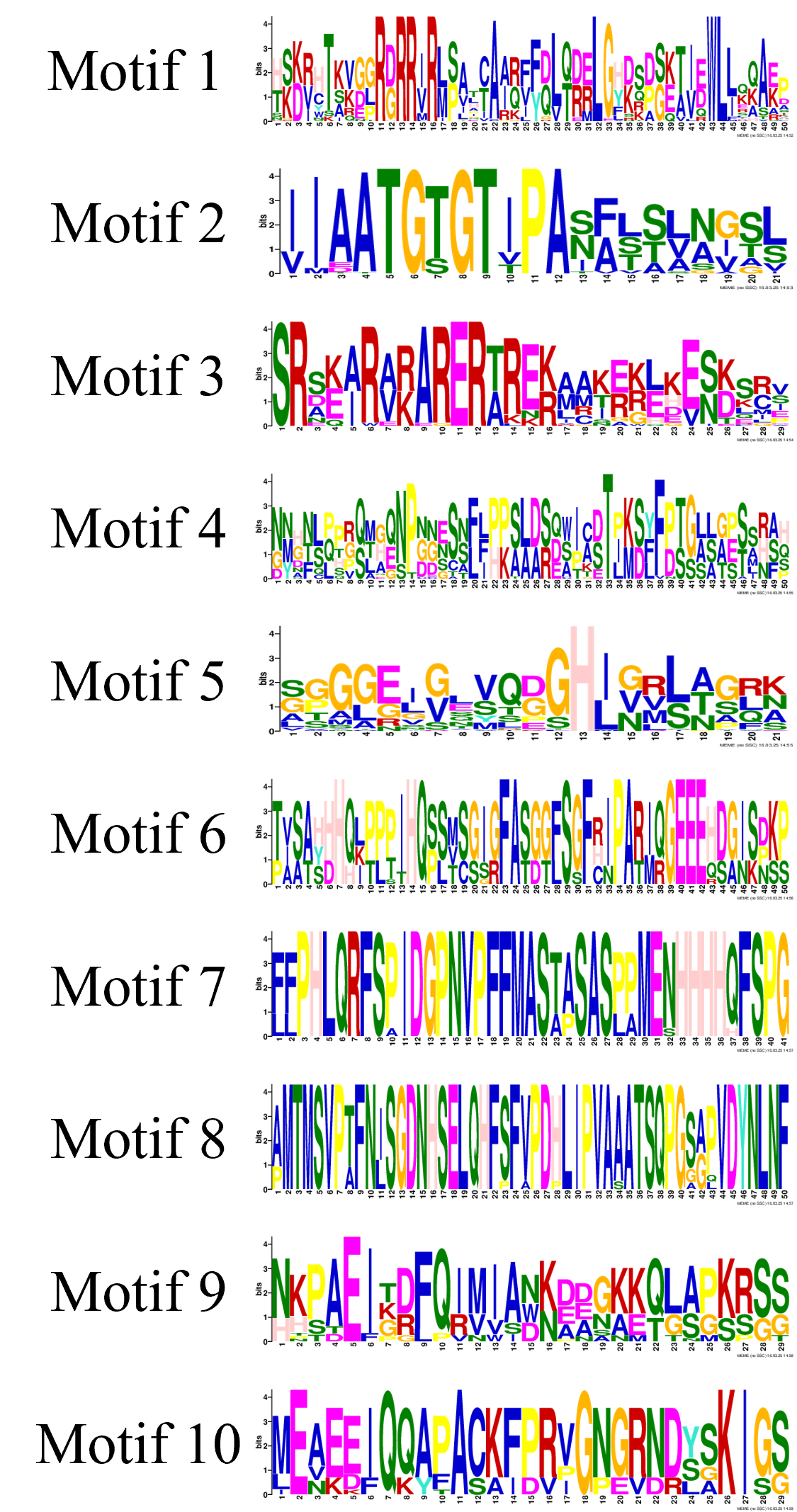

Supplement: Supplementary file 1 [file biology-14-01529-s001.zip › Figure S2.Motif logo analysis of the TCP gene family.tiff]
